# In-vivo carotid T_2_ mapping can accurately quantify plaque lipid content to discriminate between symptomatic and asymptomatic patients: histological validation, scan-rescan reproducibility and clinical study

**DOI:** 10.1186/1532-429X-18-S1-W10

**Published:** 2016-01-27

**Authors:** Luca Biasiolli, Joshua T Chai, Linqing Li, Ashok Handa, Peter Jezzard, Robin Choudhury, Matthew D Robson

**Affiliations:** 1 grid.4991.50000000419368948FMRIB Centre, Nuffield Department of Clinical Neurosciences, University of Oxford, Oxford, United Kingdom; 2 grid.94365.3d0000000122975165National Institutes of Health, Bethesda, MD USA; 3 grid.4991.50000000419368948AVIC Centre, Radcliffe Department of Medicine, University of Oxford, Oxford United Kingdom; 4 grid.4991.50000000419368948Nuffield Department of Surgical Sciences, University of Oxford, Oxford, United Kingdom; 5 grid.4991.50000000419368948OCMR Centre, Radcliffe Department of Medicine, University of Oxford, Oxford, United Kingdom

## Background

In-vivo carotid CMR is able to identify features of plaque vulnerability such as lipid core size. However, the current standard (multicontrast CMR) requires contrast media, extensive post-processing and subjective interpretation. Recently we proposed to use quantitative T_2_ mapping to distinguish plaque lipid from surrounding fibrous tissue and measure lipid core size. This study aimed to (1) validate plaque lipid quantification by T_2_ mapping against histology and (2) investigate if it could discriminate between symptomatic and asymptomatic patients.

## Methods

### CMR

**Figure 1:**
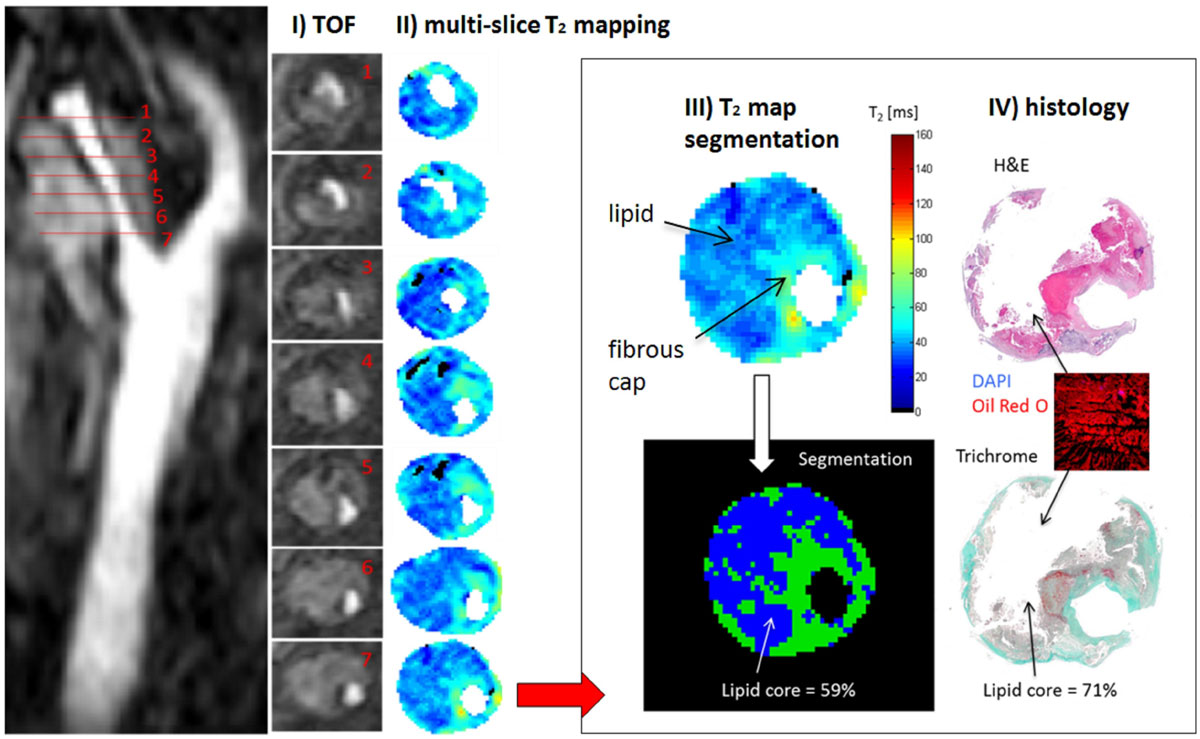
**Acquisition and analysis pipeline for plaque lipid quantification**. I) Time-of-Flight (TOF) bright-blood images were acquired to localize carotid bifurcation and stenosis. II) Multiple T_2_ map slices were acquired using our novel DANTE-MESE sequence that combines black-blood preparation based on non-selective DANTE pulse trains with Multiecho Spin-Echo. T_2_ maps were generated using nonlinear fitting; lumen and external carotid boundary were contoured semi-automatically; on T_2_ maps plaque calcification is shown in black, lipid in blue, normal vessel wall and fibrous tissue in light blue/green, and intraplaque haemorrhage in yellow/red. III) Plaque lipid area was automatically segmented on carotid T_2_ maps using leave-one-out cross-validation. IV) Plaque lipid area was manually segmented (blinded to T_2_ maps) on matching histological sections stained with H&E and Masson Trichrome, with Oil-red-O used to confirm lipid distribution.

40 patients scheduled for carotid endarterectomy (50-99% stenosis on Ultrasound), either symptomatic or asymptomatic, were imaged at 3T (Siemens Verio) max 24 h before surgery (IRB approved, written consent obtained). We used our novel black-blood Multiecho Spin-Echo sequence for T_2_ mapping (DANTE-MESE) to acquire 5 slices in 4 min (TR = 2 s, TE = 9-18-…-126 ms, partial Fourier = 5/8, FOV = 128 × 128 mm, matrix size = 384 × 384, slice thickness = 2 mm, slice gap = 2 mm). T_2_ maps were generated using nonlinear fitting; lumen and external carotid boundary were contoured semi-automatically; lipid core was segmented from other plaque components by T_2_ thresholding; and the optimal segmentation (i.e. highest correlation with histology) was automatically calculated using leave-one-out cross-validation (Figure 1).

### Histology

Plaques were collected at the time of carotid endarterectomy, processed and cut at 1 mm intervals using carotid bifurcation and T_1_w images as references to match T_2_ map locations. Plaque lipid area was manually segmented on sections stained with H&E and Masson Trichrome, with Oil-red-O used to confirm lipid distribution.

## Results

**Figure 2:**
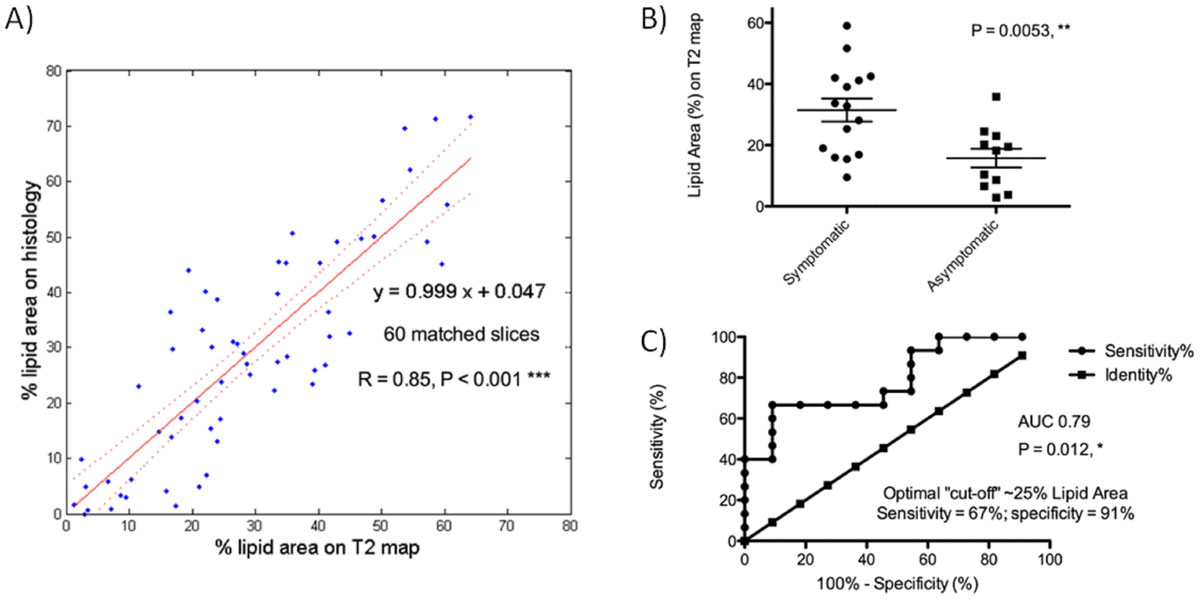
**Histological validation and clinical results**. A) The highest correlation (R = 0.85, P < 0.001) between plaque lipid area (%) measured on T_2_ maps and on histology was achieved by the thresholding combination T_2_ < 42 ms and T_2_ > 90 ms (using leave-one-out cross-validation). B) Symptomatic plaques contained significant more lipid than asymptomatic plaques (31.5 ± 3.7% vs. 15.8 ± 3.1%, P = 0.005) despite similar degree of luminal stenosis (15 symptomatic patients, median age 73, stenosis on Ultrasound = 80 ± 9% vs. 11 asymptomatic patients, median age 60, stenosis = 83 ± 9%). C) ROC curve analysis showed that T_2_ mapping had a fair/good ability to discriminate between symptomatic and asymptomatic plaques (AUC = 0.79, P = 0.012). The optimal cut-off value for lipid area was ~25% (sensitivity = 67%, specificity = 91%).

26 patient scans (median age 69) had good quality and 60 slices with matching locations on plaque histology. Lipid without haemorrhage had shorter T_2_ than normal vessel wall and fibrous tissue, whereas haemorrhage infiltrating the lipid core increased T_2_. Thresholding T_2_ < 42 ms and T_2_ > 90 ms resulted in the globally optimal lipid core segmentation (highest R = 0.85 with histology, Figure 2A). We found significantly more lipid in symptomatic than in asymptomatic plaques (Figure 2B); ROC analysis showed a fair/good ability to discriminate between the 2 groups; and the optimal cut-off value ~25% agreed with histological studies that found higher risk of rupture associated with lipid core >25% of plaque area (Figure 2C). Scan-rescan reproducibility in 9 patients was excellent: ICC = 0.89 (95% CI 0.59-0.98) and CoV = 8.9%.

## Conclusions

We have demonstrated that carotid T_2_ mapping can be used to quantify plaque lipid content with good accuracy and reproducibility, and to classify plaques as symptomatic or asymptomatic based on their lipid core size. This technique can potentially be used to identify patients at risk of plaque rupture; informing decisions of stents vs. surgery; stratify for more intensive lipid treatment; and monitor response to treatment in clinical trials.

